# Serum Levels of Infliximab and Anti-Infliximab Antibodies in Brazilian Patients with Crohn's Disease

**DOI:** 10.6061/clinics/2019/e824

**Published:** 2019-04-02

**Authors:** Luis Eduardo Miani Gomes, Francesca Aparecida Ramos da Silva, Lívia Bitencourt Pascoal, Renato Lazarin Ricci, Guilherme Nogueira, Michel Gardere Camargo, Maria de Lourdes Setsuko Ayrizono, João José Fagundes, Raquel Franco Leal

**Affiliations:** I Laboratorio de Investigacao em Doencas Inflamatorias Intestinais, Servico de Coloproctologia, Faculdade de Ciencias Medicas, Universidade Estadual de Campinas (UNICAMP), Campinas, SP, BR; II Laboratorio de Sinalizacao Celular, Universidade Estadual de Campinas (UNICAMP), Campinas, SP, BR

**Keywords:** Crohn's Disease, Infliximab, Antibody, Drug Monitoring

## Abstract

**OBJECTIVES:**

The aim of this study was to evaluate the quantitative serum level of infliximab (IFX) as well as the detection of anti-infliximab antibodies (ATIs) in patients with Crohn's disease (CD).

**METHOD:**

Forty patients with CD under treatment at a tertiary center in southeastern Brazil were evaluated. Their use of infliximab was continuous and regular. We analyzed and compared the differences in the IFX and ATI levels between the patients with active CD (CDA) and those with CD in remission (CDR).

**RESULTS:**

There was no difference in the IFX level between the CDA and CDR groups (*p*>0.05). Eighty percent of all patients had IFX levels above the therapeutic concentration (6-10 μg/mL). Two (9%) of the 22 patients with active disease and four (22.2%) of the 18 patients in remission had undetectable levels of IFX. Four (66.6%) of the six patients with undetectable levels of IFX had positive ATI levels; three of these patients were in remission, and one had active disease. In addition, the other two patients with undetectable levels of IFX presented ATI levels close to positivity (2.7 and 2.8 AU/ml). None of the patients with therapeutic or supratherapeutic IFX levels had positive ATI levels.

**CONCLUSIONS:**

The undetectable levels of IFX correlated with the detection of ATIs, which was independent of disease activity. Immunogenicity was not the main factor for the loss of response to IFX in our study, and the majority of patients in both groups (CDA and CDR) had supratherapeutic levels of IFX.

## INTRODUCTION

Crohn's disease (CD) is an inflammatory bowel disease characterized by chronic transmural inflammation in the intestinal tract. The aim of the current treatments for CD, which include corticosteroid, immunosuppressant and biological agents, is not only to control disease symptoms but above all to achieve sustained control of the intestinal inflammation. The introduction of biological agents in CD treatment has historically modified the natural process of the disease by decreasing hospitalizations 1-4. Infliximab (IFX), a monoclonal anti-TNFα chimeric antibody, was the first biological therapy used in CD patients. IFX binds with high affinity to soluble TNFα in the serum and to the transmembrane form of TNFα, neutralizing its proinflammatory activity. In addition, IFX induces T lymphocyte apoptosis, epithelial barrier recovery, and the induction of intestinal fibroblast motility, facilitating the healing of lesions 5,6.

The duration of biological treatment is not defined at all, and there is still no consensus on when to suspend this treatment approach. In patients who present an incomplete response during maintenance treatment (secondary nonresponders), the dose may be adjusted or the dose range may be reduced or even switched to another class of drug 7,8; this approach has been performed empirically in the majority of Brazilian hospitals. Drug monitoring could be broadly relevant in these scenarios of nonresponse to biological therapy as well as in reducing adverse effects if high levels of serum IFX are detected 4,7,9.

There are few studies concerning biological drug monitoring in the Brazilian inflammatory bowel disease (IBD) patient population 10. Therefore, our aim was to analyze the serum level of IFX and the detection of ATIs in a prospective patient cohort at a southeastern Brazilian tertiary center and to correlate these measurements with disease activity. This approach allowed us to evaluate the usefulness of therapeutic drug monitoring in clinical practice.

## METHODS

### Patients and ethics statement

CD patients who were followed at the Clinical Hospital of the University of Campinas (UNICAMP) and were in the maintenance phase of IFX therapy were included in the study. Of the 154 patients using IFX in the IBD outpatient clinic at the time of the study, 40 patients between 18 and 70 years old were selected sequentially from March 2016 to March 2017. All patients had already received induction therapy (0, 2, 6 weeks), followed by maintenance therapy (5 mg/kg). Disease activity was assessed by colonoscopy (active disease defined as a CDEIS score ≥5 or the presence of deep ulcers in at least one intestinal segment) or nuclear magnetic resonance (NMR) enterography (active disease defined as the presence of deep ulcers in at least one intestinal segment). Patients were included in the study only if they had scheduled these complementary examinations in the period spanning from one month before to one month after the peripheral blood sample collection. The patients were separated into two groups: those with active disease (CDA group) and those with disease in remission (CDR group). Calculation of the Crohn's disease activity index (CDAI) was performed, and the C-reactive protein serum level was also obtained. Table 1 shows the clinical and demographic characteristics of the patients included in the study.

The study was conducted in accordance with the Declaration of Helsinki and was approved by the Ethics Committee of the University of Campinas (CAAE n° 53097116.2.0000.5404). Each participant read and signed a written consent form. The laboratory analysis was carried out at the IBD Research Laboratory of the School of Medical Sciences, University of Campinas (UNICAMP).

### Enzyme-linked immunosorbent assay (ELISA)

Peripheral blood samples were collected just before the new maintenance infusion and centrifuged at 3,500 rpm for 15 minutes at 4°C, and the serum aliquots were snap-frozen and stored at -20°C. The serum samples were kept at room temperature only during the ELISA analysis. The IFX and ATI serum levels were detected using a quantitative ELISA from Promonitor^®^ (Progenika Biophama, S. A. Spain) according to the manufacturer's instructions.

The detection level of the IFX test was 0.035 to 14.4 μg/ml, while the adopted therapeutic range was 6 to 10 μg/ml. Concerning the test for the presence of ATIs, it was considered positive when the level detected was >5 AU/mL, in accordance with the manufacturer's instructions.

### Statistical analysis

All results are reported as the mean ± SEM. The *Kolmogorov*-*Smirnov test* was used to investigate whether the data followed a normal *Gaussian* distribution (*p*>0.1). The data were analyzed using the nonparametric Mann-Whitney Test. Binomial logistic regression analysis was performed for correlations between the IFX/ATI levels and clinical activity. The level of significance was set at *p*<0.05.

## RESULTS

### CD patients with active disease and those with CD in remission exhibited similar serum levels of infliximab

**Figure 1:**
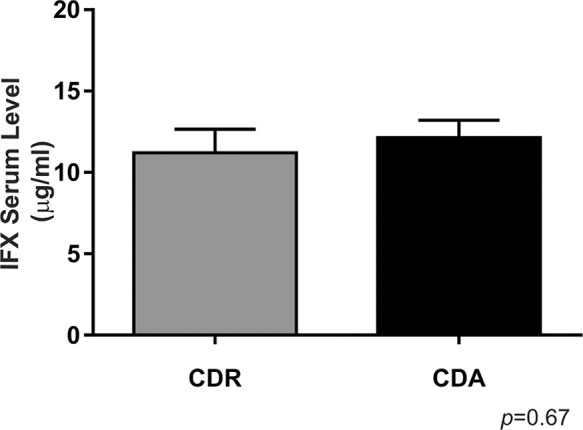
Infliximab serum levels in Crohn's disease patients with active disease and those in remission. For CDR, *n*=18; for CDA, *n*=22. There was no significant difference between the groups. CDR: Group of patients with Crohn's disease in remission; CDA: Group of patients with active Crohn's disease.

No differences in the level of IFX could be observed when comparing the patients in the CDA and CDR groups (*p*>0.05). Eighty percent (80%) of all patients had IFX levels above the therapeutic concentration (6-10 μg/mL), and of these patients, 18 had active disease and 14 were in remission. Two (9%) of the 22 patients with active disease and four (22.2%) of the 18 patients in remission had undetectable levels of IFX (Figure 1). There was no correlation between the IFX and ATI serum levels and disease activity (Table 2).

### Immunogenicity was not the main cause of the loss of response after IFX therapy

To investigate treatment immunogenicity, we performed an ELISA to detect anti-drug antibodies in the peripheral blood of all patients included in the study. ATI levels >5 AU/ml were detected in only four (10%) patients; of these four patients, one belonged to the CDA group and three belonged to the CDR group.

**Figure 2:**
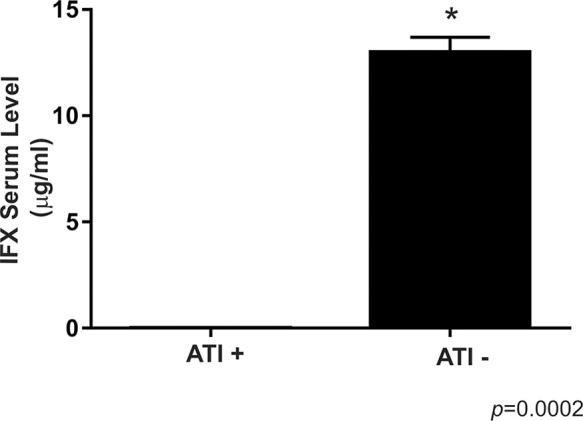
Comparison between the infliximab serum levels of Crohn's disease patients with positive and negative levels of anti-infliximab antibodies. For ATI+, *n*=4; for ATI -, *n*=36. The symbol * indicates a significant difference (*p*<0.05) between the groups, ATI- *versus* ATI+. ATI: anti-infliximab antibody.

Four (66.6%) of the six patients with undetectable levels of IFX had positive ATI levels, and three of these patients were in remission, while one had active disease. In addition, the two other patients with undetectable levels of IFX (one in remission and one with disease disease) presented with ATI levels close to the positivity threshold (2.7 and 2.8 UA/ml). All patients with positive ATI levels underwent combined therapy with immunosuppressants. None of the patients with therapeutic or supratherapeutic levels of IFX had positive ATI levels. Figure 2 illustrates these findings.

### Combined therapy with immunosuppressants did not affect the IFX levels of the patients with active disease or those of the patients in remission

**Figure 3:**
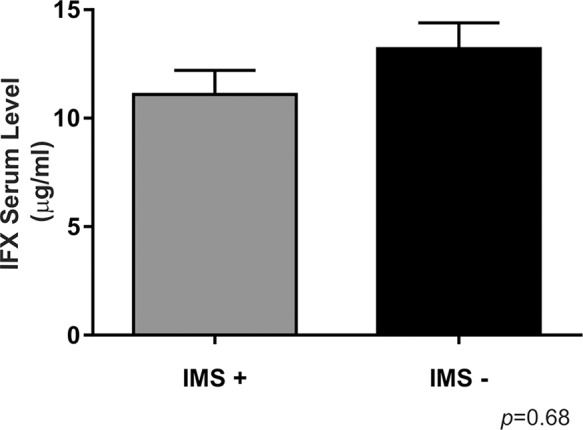
Infliximab serum levels compared between Crohn's disease patients stratified by their concomitant immunosuppressant use. For IMS+, *n*=28; for IMS -, *n*=12, *p<0.05 is considered statistically significant versus the IMS+group. IMS: immunosuppressant.

Combination therapy involving immunosuppressants was used by 28 (70%) patients. There was no significant difference in the IFX levels according to immunosuppressant use (*p*>0.05) (Figure 3).

## DISCUSSION

Therapy with IFX is commonly used in patients with CD to achieve a clinical response and to maintain sustained remission. Although most of our patients had supratherapeutic levels of IFX, all patients with positive or close to positive ATI levels had undetectable levels of the drug, regardless of the endoscopic or radiological CD activity status. There was no correlation with CD activity in our results. However, the levels of IFX and ATIs correlated with each other (all patients with undetectable levels of IFX presented positive or close to positive ATI levels) and may be relevant in patients with active disease to improve the therapeutic management and outcomes of CD. Indeed, a consensus has not been reached in the literature regarding the association between the drug levels and activity of CD. Both negative and positive associations have been found, and these contrasting results depend on the anti-TNFα agent evaluated (adalimumab or infliximab) 11-14. More data are needed to explain the variation in the drug levels. In a recent retrospective study that evaluated 76 patients with IBD (72% with CD) who lost responsiveness to IFX, clinical improvement was observed after increasing the IFX dose regardless of the IFX serum concentration 15. Although this study could not confirm the usefulness of drug monitoring, other studies have revealed that this monitoring is relevant to anticipating the sustained long-term outcome of IFX therapy and to improving efficacy and decreasing the risk of adverse events during the maintenance phase of treatment 16-21. Recently, Travis et al. 22 reported that the implementation of uniform practices to optimize IFX therapy should include a standardized drug monitoring approach with a goal of producing IFX levels greater than 5 μg/mL. Moreover, when the IFX concentration after week 14 and the ATI levels were added to the clinical data, dose and/or range changes based on actual dosing were recommended in 48/50 (96%) patients 23. Brandse et al. 24 showed that IFX levels below 3 μg/mL increase the risk of developing ATIs. The identification of influential pharmacokinetic factors and ATIs improves the prediction of IFX levels, potentially making individualized dosing and cost reduction possible 19,25,26.

We did not find any difference in the IFX levels dependent on immunosuppressant use (Figure 3), which agrees with the findings of other studies in the literature, such as the study by Oh et al. 14. The patients who received a concomitant immunosuppressant tended to have a lower incidence of antibody formation than those who did not receive a concomitant immunosuppressant, as reported by Buurman et al. (10% *vs* 26%, respectively), but the difference was not significant 27. The effect of immunosuppressants may be more important in patients treated with episodic IFX (0% *vs* 60%, respectively, *p*<0.018) than in patients receiving scheduled maintenance treatment (13% *vs* 15%, respectively, *p*=9) 27.

Regarding the ATI levels, the available data suggest that the presence of ATIs may have a negative impact on the clinical outcome, although this effect is not absolute 28. In a retrospective study, the presence of ATIs reduced the likelihood that IFX intensification would restore the clinical effect of IFX in patients with secondary loss of response 29. Thus far, measuring IFX levels along with ATI levels has been suggested to be useful in patients with loss of secondary response. Afif et al. 29 showed that in patients with subtherapeutic concentrations of IFX and negative ATI levels, IFX dose escalation was superior to switching to another biological compound. In contrast, ATI positivity does not affect the rate of clinical remission, endoscopic improvement or C-reactive protein (CRP) level in CD patients under long-term infliximab therapy 18. Although our data indicate a low number of patients with positive ATI levels, we believe that the interpretation of the ATI level status should be made with caution. In fact, there is also evidence that the concentration of ATIs may fluctuate and even drop below detectable levels after infliximab intensification 30. Moreover, negative ATI levels are possible in the presence of higher serum levels of IFX. In addition, the ATI assay measures the serum levels of free ATIs but lacks sensitivity towards IgG4 because only the bivalent fraction is detected 31,32. However, the relevance of these data lies in showing how the detected ATI levels may affect clinical practice. Patients with positive ATI levels usually have undetectable drug levels, and this clinical situation can be better classified as an immunogenic nonresponse. The practical recommendation is that ELISA ATI levels should be measured not in all patients but mainly in those patients with undetectable IFX levels.

The serum concentration of IFX during scheduled maintenance therapy predicts the patient clinical outcome. Factors such as the formation of antibodies, pharmacokinetics and the albumin level may modulate serum IFX and, consequently, the clinical response to the therapy 27. The impact on the development of antibodies was minimal in our patients receiving regularly scheduled infliximab treatment. Therefore, decreasing the IFX dose would result in a lower cost and optimization of the drug for the majority of our patients, and it would be a possible strategy to employ.

The main limitation of this study was the lack of longitudinal data for the measurement of the IFX and ATI levels over time and over the course of the disease 33. However, our cross-sectional findings were very useful for obtaining an overview of biological therapy monitoring in our center. The main unexpected finding was the high number of patients with supratherapeutic levels of IFX. In our institution, the issue as to whether it is safe to reduce the dose of the drug in these patients, considering the risk of recurrence of the disease, will need to be discussed. Moreover, immunogenicity was not the main cause of loss of response after IFX therapy in our patients, as the minority of the CDA group presented positive ATI levels, but a correlation with the undetectable levels of IFX was detected. The main cause of continued disease in our patients with adequate or supratherapeutic serum levels of IFX with negative ATI levels was probably the development of other proinflammatory pathways that do not depend on TNF-α.

In summary, the introduction of drug monitoring for anti-TNFα agents, including drug level and ATI detection, may allow for more personalized therapeutic management with better dose adjustment or even a change to another drug with a different action mechanism. Furthermore, this may reduce expenses associated with medication, mainly in the patients with supratherapeutic drug levels.

## AUTHOR CONTRIBUTIONS

Leal RF designed the study. Gomes LEM, Silva FAR and Ricci RL collected the data. Leal RF, Ayrizono MLS and Camargo MG recruited the patients. Gomes LEM, Nogueira G and Silva FAR carried out the experiments. Pascoal LB was responsible for the statistical analysis. Leal RF and Gomes LEM wrote the manuscript. Leal RF, Pascoal LB and Fagundes JJ contributed to the final manuscript review. All authors approved the manuscript’s submission.

## Figures and Tables

**Table 1:** Demographic and clinical characteristics of the patients included in the study.

	CDA Group	CDR Group
Number of patients	22	18
Gender (M/F)	12/10	10/8
Age (years)	35.5 [19-59]	42.5 [18-61]
Disease duration (months)	84 [8-360]	102 [2-300]
Age at diagnosis (A1/A2/A3)*	3/18/1	2/12/4
Location (L1/L2/L3/L4)*	8/12/2/0	9/9/0/0
Behavior (B1/B2/B3)*	15/5/2	15/1/2
Perianal disease (yes/no)	12/10	8/10
Immunosuppressant use (yes/no)	16/6	10/8
Duration of anti-TNFα therapy (months)	24 [2-168]	27 [2-192]
IFX interval adjustment (yes/no)^∞^	8/14	4/14
Previous anti-TNFα therapy (yes/no)	5/17	5/13
CDAI score	210.6 [51.6-572.2]	141.5 [53.8-497.5]
CDEIS score	9.6 [4.25-22.4]^#^	0 [0-3]^#^†
Signals of inflammation in the NMR (yes/no)^φ^	8/0	0/4
Albumin level (g/dL)	3.4 (2-4.9)	3.6 (2.3-5)
CRP level (mg/L)	11.2 (0.09-103)	3.31 (0.37-36.8)

Numerical variables are described as the median [min, max], and categorical variables are described as absolute frequencies. *Montreal Classification. **^∞^**Patients whose IFX administration interval was adjusted to 4 or 6 weeks and who remained under this drug regimen for at least 6 months before their blood was collected. **^#^**CDEIS scores were calculated for 14 patients in each group. **^φ^**Presence of ulcers and mucosal enhancement in at least one intestinal segment. ^†^*p*<0.0001 compared with the CDA group. CDA = active Crohn’s disease. CDR = Crohn’s disease in remission. M = male. F = female. TNF = tumor necrosis factor. CDAI = Crohn’s Disease Activity Index. CDEIS = Crohn’s Disease Endoscopic Index of Severity. NMR = Nuclear Magnetic Resonance. CRP = C-reactive protein.

**Table 2:** Numerical data from the logistic regression analysis of the correlation between the IFX (infliximab) or ATI (anti-infliximab antibody) serum levels and disease activity.

	IFX level (µg/ml)	ATI level (AU/ml)
*p* value	*p*>0.05	*p*>0.05
Odds ratio (95% CI)	0.429 (0.04-4.578)	0.111 (0.005-2.727)
